# Pyrolysis and Combustion Kinetics of Garden Waste Pellets as Solid Biofuel for Thermochemical Energy Recovery

**DOI:** 10.3390/ma18071634

**Published:** 2025-04-03

**Authors:** Jonatan Gutiérrez, Juan F. Pérez

**Affiliations:** Grupo de Manejo Eficiente de la Energía—GIMEL, Departamento de Ingeniería Mecánica, Facultad de Ingeniería, Universidad de Antioquia—UdeA, Calle 70 No. 52-21, 050010 Medellín, Colombia; jonatan.guierrez@udea.edu.co

**Keywords:** fallen leaf pellets, pyrolysis, combustion, reactivity, thermogravimetric analysis, kinetic analysis

## Abstract

The fallen leaf has the potential to be energy-valorized in cities with sustainability goals. Thermochemical characterization of garden waste through pyrolysis and combustion kinetics will establish the reactivity of this lignocellulosic biomass as biofuel for thermochemical conversion processes for energy recovery. Herein, the thermal degradation of two types of pellets produced from fallen leaf (pellets without glycerol PG0, and pellets with 5 wt% glycerol PG5) are characterized under inert and oxidative atmospheres using three different approaches: thermogravimetry (TG) and differential thermogravimetry (DTG) analyses, TG-based reactivity, and reaction kinetics from three model-free isoconversional methods. The model-free isoconversional methods are Flynn–Wall–Ozawa (FWO), Kissinger–Akahira–Sunose (KAS), and Friedman, which were applied for estimating the kinetic parameters, activation energy (Eα) and pre-exponential factor, using different heating rates (20, 30, and 40 °C/min) to ensure reliable data interpretation. The pyrolysis results showed that PG5 was more reactive compared to PG0 because the addition of glycerol during the pelletizing process increased the volatile matter and oxygen content in PG5. Likewise, the higher reactivity of PG5 under pyrolysis was determined by average activation energy (Eα) with an average value of 96.82 kJ/mol compared to 106.46 kJ/mol for PG0. During the combustion process, Eα was 90.70 kJ/mol and 90.29 kJ/mol for PG0 and PG5, respectively. Finally, both materials exhibited higher reactivity under an oxidative atmosphere. Therefore, according to our results, the pellets produced from leaf litter can be used as biofuels for thermochemical processes, highlighting that using glycerol as a binder favors the reactivity of the densified garden waste.

## 1. Introduction

One of the greatest challenges facing the world’s population is managing increasing municipal solid waste (MSW). According to the World Bank, global MSW production will increase from 2 × 10^9^ tons in 2016 to ~3.4 × 10^9^ tons by 2050 [1]. This increase in MSW is directly related to the population growth in urban areas and economic development. Around 56% of the global population (~4400 million inhabitants) was in urban areas in 2022 [2,3]. In total, 38% of the total MSW produced worldwide corresponds to dry recyclable products, while 44% represents food and garden waste [1]. Garden waste includes fallen leaves from trees, which can be utilized through composting, digestion for biogas production, biochar, and biohydrogen [4,5], and through thermochemical processes such as gasification, pyrolysis, and combustion for energy recovery [6]. When leaf litter is used in energy generation processes instead of composting, CO_2_ emissions are reduced by 15% to 70% [7,8]. In addition, seeking to reduce CO_2_ emissions, the thermochemical conversion of fallen leaf trees provides an alternative solution to the growing energy demand and environmental pressures (climate change and air pollution) caused by the use of fossil fuels [9]. The most common thermochemical conversion technologies include pyrolysis, gasification, and combustion. The proper selection and operation of biomass thermochemical conversion technology require understanding and quantifying the kinetic behavior of biomass [10]. This analysis contributes to increasing the efficiency of the energy valorization process, extending the operation time of reactors, and minimizing pollutant emissions [11].

The kinetic behavior characterization of the biomass as a solid biofuel can be carried out through thermogravimetric analysis (TGA), under isothermal or non-isothermal conditions, coupled with model-free methods [12]. TGA provides the relationship between temperature and the physicochemical properties of fuels during pyrolysis and combustion [9], as well as reactivity properties by differential thermogravimetry analysis (DTG) [13]. The kinetic parameters of pyrolysis and combustion can be determined from thermogravimetric data at different heating rates using isoconversional mathematical models, known as model-free methods, such as the Kissinger–Akahira–Sunose (KAS), Flynn–Wall–Ozawa (FWO), and Friedman models [9,14]. Model-free methods are frequently used in biomass kinetic characterization because their associated error is lower than the model-fitting methods [15,16].

At the beginning of the last decade, fallen leaves were characterized as a potential solid biofuel. The higher ash content of fallen leaves (20 times higher) diminished their heating value regarding firewood between 11.7% and 15.1% [17]. The assessment of kinetic properties under pyrolysis was carried out by Shi et al. [18], analyzing fallen leaves and waste tires under model-free methods like KAS and FWO. The fallen leaves’ activation energy was 153.17 kJ/mol for KAS, and 155.15 kJ/mol for FWO, while the preexponential factor varied between 1.02 × 10^7^ and 2.34 × 10^7^. The authors highlighted that the mixture of these wastes could be used as an energy feedstock. Mudryk et al. [19] pelletized and characterized fallen leaves from different tree species as fuel. The densified materials reached good properties as fuel, such as heating value (15 MJ/kg), bulk density (600–660 kg/m^3^), durability (90–96%), and moisture content (10–12.5%). Furthermore, according to the ash melting point of the pellets (1310–1430 °C), the authors stated that the ash removal systems can work without ash melting problems. Nevertheless, Montiel and Pérez [20], reported that the ash melting point of pellets’ fallen leaves varies by ~100 °C, between 1226 °C and 1334 °C.

Senneca and Cerciello [21] reported in their review that the combustion kinetic parameters by TGA of different fallen leaf trees (apple, tea, and trees) varied between 20.6 and 230 kJ/mol for the activation energy and from 2.65 × 10^0^ to 6.7 × 10^26^ for the preexponential factor. These variations are mainly ascribed to the structural complexity and various extractives in the biomass, which lead to multiple overlapping components. Tin et al. [10] highlighted the feasibility of pyrolysis or gasification of fallen leaves for hydrogen production due to their high cellulose content. In this case, the estimation of the thermochemical kinetic rates (activation energy and pre-exponential factor) is required for simulating the conversion process of fallen leaves to hydrogen. The kinetics of pyrolysis and combustion have been studied to determine different biomass types’ activation energy and pre-exponential factor [11,13,14]. In general, using N_2_ or air as carrier gases for pyrolysis and combustion kinetics, respectively [12,22], and considering at least three different heating rates [11,12,23]. Wu et al. [12] studied the kinetics of pyrolysis and combustion for cattle manure, finding that the reactions for both thermochemical processes occur in four steps. The reported activation energy values were 93.63 kJ/mol and 84.53 kJ/mol for the first two reaction zones in pyrolysis, and 83.03 kJ/mol and 55.65 kJ/mol for the first two reaction zones in combustion. On the other hand, Ashraf et al. [24] reported that the thermal decomposition of cattle manure occurs in three phases: phase I (dehydration), phase II (devolatilization), and phase III, which is carbonization for pyrolysis, while for combustion, this phase is defined as carbon oxidation. The average reported activation energy was 146 kJ/mol and 127 kJ/mol for pyrolysis and combustion, respectively.

Other studies have focused on obtaining the kinetics of pyrolysis and combustion using isoconversional methods for biomass such as corn straw, wood chips, and rice husk, with activation energies between 70 kJ/mol–100 kJ/mol for pyrolysis (drying, pyrolysis, and carbonization), and 140 kJ/mol–180 kJ/mol for combustion (drying, devolatilization, and carbon combustion) [13]. The isoconversional TGA methods approach has also been applied to cereals (*Penissetum glaucum* (L.) R. Br.). Its activation energy under pyrolysis was 89.63 kJ/mol and 83.89 kJ/mol, calculated by the FWO and KAS methods, respectively. While under combustion, the activation energy reached lower values, 57.27 kJ/mol (FWO) and 49.47 kJ/mol (KAS) [14]. The kinetics of pyrolysis and combustion of pine wood [9,25], seeds, leaves, and date palm stems have also been analyzed, with activation energies ranging from 9.7 kJ/mol to 43.6 kJ/mol for pyrolysis, and from 9.04 kJ/mol to 30.95 kJ/mol for combustion [26].

Although fallen leaves have been characterized in digestion processes for electricity production (234 kWh/t_leaves_), garden waste has potential as a raw material for thermochemical processes [6,27,28]. The literature review revealed that the pyrolysis and combustion kinetics of pellets made from fallen tree leaves have not yet been studied. Therefore, the gap to be addressed herein is based on the study of the pyrolysis and combustion processes of fallen leaf pellets, providing a holistic view of the reactivity of the waste garden biomass. The thermal degradation of the pellets under inert and oxidative atmospheres is characterized using three different approaches: First, thermogravimetry (TG) and differential thermogravimetry (DTG) analyses; second, TG-based reactivity, and third, the reaction kinetics from three model-free isoconversional methods. The reactivity and the kinetic parameters can be used for technology selection, design, and optimization of reactors used in the energy valorization of the waste garden biomass [6]. As a result, fallen leaves as a solid biofuel source have the potential to be valorized in cities with sustainability goals within the framework of the United Nations Sustainable Development Goals [29].

## 2. Materials and Methods

The behavior of garden waste (fallen leaf) pellets under pyrolysis and combustion regimes is characterized to evaluate their reactive potential as solid biofuel in thermochemical processes. Pellets, made in a previous work without (PG0) and with glycerol (PG5) as a binder [6], are considered to determine their effect on the reactivity of the biofuels. Then, both pellet types were physicochemical and energetically characterized by proximate and ultimate analyses, bulk and particle densities, Scanning Electron Microscopy (SEM), calorific value, and Differential Thermogravimetric Analysis (DTG). Subsequently, kinetic parameters (pre-exponential factor and activation energy) were determined using model-free methods [9,14]. It is worth mentioning that the measurements were carried out twice for the properties where the standard deviation is not reported, and the average value is presented.

### 2.1. Biomass Samples

The biomass characterized in this work corresponds to leaf litter pellets from the central campus of the University of Antioquia (UdeA)—Colombia. The campus has an extension of ~24 hectares, of which 34% are gardens, with ~2280 trees planted, classified into 154 species, representing a forest density of ~280 trees/ha-garden. These trees produce an average of ~2.8 tons of dry leaf litter per month [27]. In addition, the UdeA main campus is located in the new northern zone of Medellín, which includes the Parque Norte (~2.8 t/month of leaf litter) and the city’s botanical garden (~3.5 t/month of leaf litter). In total, the new northern zone from Medellín generates about 9 t/month of waste garden (leaf litter) that can be valorized through thermochemical processes such as pyrolysis, gasification, or combustion for energy production (electricity and/or heat) seeking to reduce greenhouse gas emission as part of the sustainable university campus strategy [30].

The leaf litter generated at UdeA was pelletized to be used as a solid biofuel because this pretreatment improves several properties such as energy density, which was 2800 MJ/m^3^ for non-pretreated waste, and 5500–7300 MJ/m^3^ for pelletized one [6]. The pelletizing process was carried out and described in detail by Gonzáles et al. [6], who used a roller pelletizer with a matrix with holes of 8 mm in diameter (power machine 3.75 kW, and capacity of 35 kg/h). The ground leaf litter with a moisture content of ~10 wt% was fed into the pelletizer to produce two types of pellets. One pellet without (PG0) and the other one with 5 wt% of glycerol as a binder (PG5). The physicochemical and energy properties of the two pellet types, adapted from our previous works, are presented in Table 1 [6,28]. The properties of the wood pellets (WP) were taken from Gutiérrez et al. [25] to be used as a benchmark for comparison.

The WP refers to pellets made from Pinus Patula wood biomass, which possesses high dendroenergy potential in Colombia. Its silvicultural properties include a planted area in the country of approximately 38,500 ha, an annual yield of 20 m^3^/ha-year, and a harvest time of 13 years [31].

**Table 1:** Physicochemical and energy properties of PG0, PG5, and WP.

Properties	Standard	Biomass		
		**PG0**	**PG5**	**WP [25]**
Ultimate analysis [wt% dry base and ash free]				
C	ASTM D5373-08 [32]	51.7 ± 0.5	49.7 ± 0.1	47.0
H	ASTM D5373-08	7.1 ± 0.2	6.9 ± 0.1	5.7
O	By difference	41.2 ± 0.6	43.4 ± 0.1	47.3
Proximate analysis [wt% dry base]				
Volatile matter (VM)	ASTM D5142-04 [33]	69.0	72.1	84.6
Fixed carbon (FC)	By difference	10.3	9.9	14.1
Ash	ASTM D5142-04	20.6	18.0	1.3
Moisture [wt%]	ASTM D5142-04	5.0 ± 0.1	5.2 ± 0.2	7.9
Physical properties				
Bulk density [kg/m^3^]	ASTM E873-82 [34]	524.7 ± 5.6	461.5 ± 9.0	560.0
Particle density [kg/m^3^]	Oil immersion	1102.1 ± 32.2	980.2 ± 8.7	1153.6
Packing factor [-]	-	0.5	0.5	0.5
Energy properties				
LHV (dry base) [MJ/kg]	ASTM D2015 [35]	13.5 ± 0.1	14.0 ± 0.1	19.0 ± 0.1
Fiber composition (wt%)				
Lignin ^a^	-	29.4	27.1	43.7 ^b^
Cellulose ^a^	-	32.2	24.1	32.8 ^b^
Hemicellulose ^a^	-	36.7	37.5	12.7 ^b^

^a^ Fiber analysis determined by deconvolution of the DTG curve [6]. ^b^ Fiber analysis measured by the Van Soest method AOAC 962.09 y 978.10 [25].

### 2.2. Reactivity by Thermogravimetric Analysis

Reactivity analysis under pyrolysis and combustion of the biomass samples (PG0 and PG5) was performed using a TGA Q50 thermobalance. The procedure consisted of drying the biomass samples at 105 °C for 24 h before analysis. Before the drying process, pellets were cut with a scalpel to preserve their properties, and samples of ~10 mg were taken for each analysis. For the reactivity analysis, the dry samples of both biomasses were heated from 25 °C to 900 °C with a heating rate of 20 °C/min. The experiments were performed under two types of atmospheres, inert (N_2_) and oxidative (air), with a fixed flow rate of 120 mL/min. The reactivity analysis allows for determining the reaction rates, and the thermal stability under devolatilization and oxidation conditions for the biomass samples.

The reaction rate is calculated by Equation (1) and the thermal stability was determined with the base temperature (T_b_), which corresponds to the temperature where the derivative (DTG) is equal to 1%/min during the devolatilization stage [36]. In Equation (1), the term $R_{a}$ (min^−1^) corresponds to the reactivity, *m*_0_ (mg) is the initial mass of the sample subjected to thermogravimetric analysis, and *DTG_max_* (mg/min) is the maximum value of the DTG curve [37].

(1) $$R_{a}=\frac{1}{m_{0}}\cdot {DTG}_{max}$$

### 2.3. Kinetic of Pyrolysis and Combustion

The kinetics of biomass thermal decomposition under an inert or oxidative atmosphere can be accurately studied by thermogravimetric analysis combined with some empirical models, either model-free isoconversional or model-fitting methods. Herein, three model-free isoconversional methods were used because they are more reliable in calculating the activation energy and the pre-exponential factor regarding the model-fitting methods [9,13,15]. Through the results of kinetic parameters, it is possible to understand the thermal degradation of the biomass samples (PG0 and PG5) for the selection, design, and optimization of thermochemical conversion technology, and even the implementation of mathematical models of the thermochemical process [9,12].

The kinetic parameters used to determine the reactivity of the biomass, under inert and oxidative atmospheres, are the activation energy (E_α_, kJ/mol) and the pre-exponential factor (A_α_, 1/s). The kinetic analysis is based on model-free methods by combining experimental tests at different heating rates [20,38]. Experimental tests were performed on a TA Instruments TGA Q50 thermobalance. About 10 mg of the cut pieces of pellets (PG0 and PG5) were used in each test. The total volumetric gas flow rate supplied was 120 mL/min. Air was used for the oxidative atmosphere tests and N_2_ for the inert atmosphere tests. For each type of atmosphere, the samples were subjected to thermal degradation from 25 °C to 900 °C, and the heating rate was set up at 20, 30, and 40 °C/min. At the maximum temperature, an isotherm was carried out for 15 min. This method was adapted from Wu et al. [12] and Broström et al. [22]. The three heating rate values considered here were selected because they represent the phenomena of the biomass thermochemical processes [39,40]. The Kinetics Committee of the International Confederation for Thermal Analysis and Calorimetry (ICTAC) stated that analysis with at least three different heating rates can provide reliable kinetic data [41]. For each biomass sample (PG0 and PG5), the variation of the mass conversion fraction (pyrolyzed/oxidized) (α) (Equation (2)) was calculated and plotted as a function of the heating rates [24,42].

(2) $$\alpha =\frac{m_{0}-m_{t}}{m_{0}-m_{\infty }}$$

where $m_{0}$ (mg) corresponds to the initial mass of the sample, $m_{\infty }$ (mg) is the final mass of the sample, and $m_{t}$ (mg) refers to the mass of the sample at a time *t*. The mass conversion fraction (α) was plotted as a function of temperature, for PG0 and PG5, to determine the ranges corresponding to each phase of the mass conversion fraction for pyrolysis and combustion. Three phases were found and defined (Section 3.2) for the pyrolysis and combustion kinetics tests carried out here. The phases correspond to phase I—dehydration, phase II—devolatilization, and phase III—carbonization [24].

The kinetic parameters calculation, activation energy (E_α_, kJ/mol), and pre-exponential factor (A_α_, 1/s), were performed in the α_i_ ranges for pyrolysis and combustion according to Flynn–Wall–Ozawa (FWO), Kissinger–Akahira–Sunose (KAS), and Friedman methods [16,43], described in Equations (3–5), respectively. The calculation of the activation energy ($E_{\alpha }$ [kJ/mol]) was performed using Equation (6) FWO method, Equation (7) KAS method, and Equation (8) Friedman method. According to the isoconversional principle, the reaction rate of thermal decomposition of biomass is a function of temperature at a given conversion rate [13]. Under this approach, isoconversional lines were plotted for each conversion rate range (α_i_) and model-free method.

(3) $$\ln\left(\beta_{j}\right)=\ln\left(\frac{A_{\alpha }E_{\alpha }}{Rg\left(\alpha \right)}\right)-5.331-1.052\frac{E_{\alpha }}{RT_{\alpha_{i}}}$$

(4) $$ln\left(\frac{\beta_{j}}{T_{\alpha_{i}}^{2}}\right)=ln\left(\frac{A_{\alpha }R}{E_{\alpha }g\left(\alpha \right)}\right)-\frac{E_{\alpha }}{RT_{\alpha_{i}}}$$

(5) $$ln\left(\beta_{j}\frac{d\alpha }{dT}\right)=ln\left(A_{\alpha }f\left(\alpha \right)\right)-\frac{E_{\alpha }}{RT_{\alpha_{i}}}$$

(6) $$E_{\alpha }=-\frac{m_{fwo}RT_{\alpha_{i}}}{1.052}$$

(7) $$E_{\alpha }=-m_{kas}RT_{\alpha_{i}}$$

(8) $$E_{\alpha }=-m_{Fried}RT_{\alpha_{i}}$$

where: $g\left(\alpha \right)$ and $f\left(\alpha \right)$ = Common reaction mechanisms in solid-state reactions [44], T_αi_ [K] = Conversion temperature at a specified α_i_, R [J/K-mol] = Universal gas constant (8.314 J/mol/K), $\beta_{j}$ [K/min] = Heating rate, $m_{fwo}$ = The slope of graph ln(β_j_) vs. 1000/T_αi_ (see Figure A2), $m_{kas}$ = The slope of graph ln(β_j_/T^2^_αi_) vs. 1000/T_αi_ (see Figure A3), and $m_{Fried}$ = The slope of graph ln(β_j_·dα_i_/dT_αi_) vs. 1000/T_αi_ (see Figure A4).

The pre-exponential factor (A_α_) of the devolatilization and oxidation reactions, for PG0 and PG5, explains the collision intensity inside the solid biofuel, which is calculated by Equation (9). This calculation methodology has been widely used in other works to determine A_α_ for different biomass types [45,46,47,48], where T_m_ (K) is the temperature corresponding to the maximum point of the plot dα/dT vs. T.

(9) $$A_{\alpha }=\frac{\beta_{j}E_{\alpha }}{RT_{m}^{2}}exp\left(\frac{E_{\alpha }}{RT_{m}}\right)$$

## 3. Results and Discussion

### 3.1. Physicochemical Characteristics of Biomass Samples

The physicochemical characteristics of PG0 and PG5 are shown in Table 1. Before characterizing the reactivity and kinetics of the pellets, their physical–chemical properties as biofuels are analyzed. It should be noted that the addition of glycerol to the pellet production process affects different properties. From the ultimate analysis, the C content was ~4% lower for PG5 than that of PG0. The hydrogen content reached a value of ~2% lower for PG5 than PG0. Concerning the wood pellets (WP), it is observed that biofuels produced from fallen tree leaves reached higher C and H contents. In contrast, the oxygen content was higher by ~13% and 8% for WP compared to PG0 and PG5, respectively. The higher oxygen content in biofuels (PG0 and PG5) decreases the stoichiometric fuel-to-air ratio during gasification or combustion processes. Therefore, biomass consumption per mass unit of air increases; consequently, PG5 is considered a more chemically reactive biofuel [27]. In the studied biomasses here, negligible contents of N nor S were detected. Therefore, using these materials as biofuels avoids the formation of NO_x_ and SO_x_ during their energy valorization through thermochemical processes. Additionally, corrosion problems are reduced due to the absence of sulfur in these solid biofuels derived from garden waste [11].

The glycerol, used as a binder, increased the volatile matter (VM) content in the pellets, whereby the fixed carbon content decreased, while the reactivity of densified fuels was enhanced due to higher volatile matter [49]. Higher VM content makes biomass feasible for the combustion process and for obtaining bio-oil by the fast pyrolysis process [26]. The VM content of PG5 was 4.4% higher than that of PG0. In contrast, the fixed carbon content of PG0 was 4.1% higher than that of PG5. Comparing the ash content of pellets produced from fallen tree leaves with WP, it is observed that the ash content is 16 and 14 times higher for PG0 and PG5, respectively (Table 1). The high ash content of the pelletized garden waste can cause deposition problems, such as fouling and slag formation [50]. Therefore, the operating temperature during the downdraft gasification of leaf litter pellets should be kept below ~1000 °C (150–200 °C lower than the initial deformation temperature, ash melting) to avoid ash agglomeration problems [20].

The bulk density of untreated leaf litter has a value of ~150 kg/m^3^ [6], while the bulk density of densified leaf litter reaches values of 524.70 kg/m^3^ and 461.5 kg/m^3^, for PG0 and PG5, respectively (Table 1). By comparing the densified biomass samples, the bulk density of PG0 is 13.7% higher than that of PG5. A lower bulk density is associated with higher reactivity during the thermochemical process, as it favors the biomass combustion rate inside the reactor [25]. The particle density is ~12% higher for PG0 than for PG5 because glycerol favors the lubricity between the ground biomass and the pelletizer matrix, inhibiting the function of lignin as a natural binder and mainly favoring mechanical interlocking [27]. The lower heating value (LHV) of PG5 is favored by the glycerol used as a binder during the densification process. Pure glycerol has an LHV of ~19 MJ/kg, while leaf litter has an LHV of 12.89 MJ/kg [27]; therefore, the LHV of the mixture tends to increase. The lower ash content also favors the LHV of PG5 [11]. Nevertheless, compared to WP, the LHV of litter pellets is lower by ~42% and ~36% for PG0 and PG5, respectively.

The fiber content for PG0 and PG5 is also presented in Table 1. The contents of hemicellulose, cellulose, and lignin, for both biofuels, are similar because they are made with the same feedstock (fallen tree leaves). Hemicellulose and cellulose are mainly associated with the content of VM, while lignin is the main contributor to biochar formation [51]. The variation in hemicellulose content for PG0 and PG5 is mild, with values of 36.37 wt% and 37.49 wt%, respectively. On the other hand, cellulose decreases with the addition of glycerol to the waste biomass, from 32.20 wt% (PG0) down to 24.05 wt% (PG5), i.e., a reduction of 34%. This reduction is attributed to the formation of structured solid bridges due to the addition of glycerol [52]. The lignin content of PG5 was 8.4% lower in comparison with PG0. Gonzales et al. [6] found a waxy biopolymer called cutin in the chemical composition of fallen leaf pellets, which is degraded between 450 °C and 500 °C. Cutin, composed of long-chain esters and other fatty acids, is an important component of the plant cuticle [6].

### 3.2. TG and DTG Analyses

**Figure 1:**
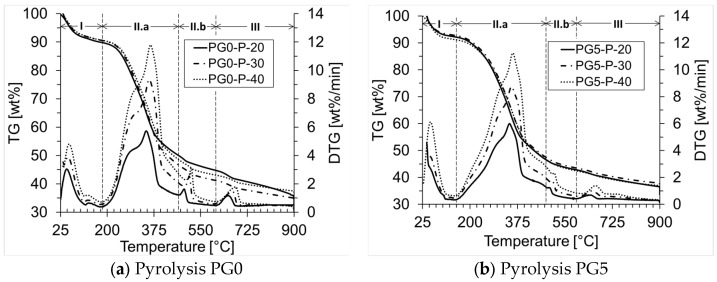

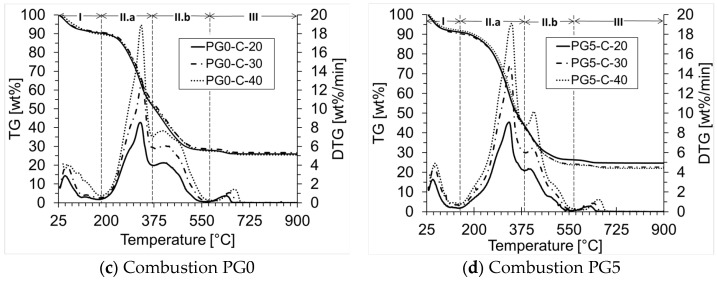
TG and DTG curves for PG0 and PG5 under pyrolysis (P) and combustion (C) in function of different heating rates.

The thermal degradation behavior of PG0 and PG5 under pyrolysis and combustion was assessed under three different heating rates (20, 30, and 40 °C/min). The TG (thermogravimetry) and DTG (differential thermogravimetry) curves for the characterized biomass samples are shown in Figure 1a–d. The profiles show similar behavior for PG0 and PG5 under pyrolysis (coded as PG0-P and PG5-P) and under combustion (PG0-C and PG5-C). This similar behavior is because both samples correspond to leaf litter pellets whose proximate analyses variation is mild (Table 1). The similarity of the plots indicates an analogous reaction mechanism under different heating rates. Consequently, the reaction temperature is mainly influenced by the atmosphere (pyrolysis and combustion) [12].

The mass loss for PG0 under an inert atmosphere exhibited three phases [53]. Phase I—drying, phase II—devolatilization, and phase III—carbonization, with two sub-stages in the devolatilization phase (Figure 1a) [12,13]. The first stage occurs from ambient temperature to ~180 °C, with a mass loss between 9.35 wt% and 10.16 wt%. This phase corresponds to the moisture evaporation from the biomass [14]. The main reaction area (phases II.a and II.b), for PG0, occurred between 180 °C and 610 °C, corresponding to the highest organic matter loss. In this area, the TG curve slope became more negative. Phase II.a occurred between 180 °C and 470 °C, corresponding to a mass loss between ~40 wt% and 44.10 wt%. In this zone (II.a), the DTG profiles showed a main peak (maximum value of the derivative) and a side peak (a shoulder to the left of the maximum peak). The main peak (highest mass loss rate) was formed by pyrolysis of cellulose at a maximum temperature of 347 °C, 358 °C, and 362 °C at heating rates of 20, 30, and 40 °C/min, respectively. The side peak was formed by pyrolysis of hemicellulose, and the separation of the two peaks is proportional to the relative content of hemicellulose and cellulose in the biomass [12,13]. For phase II.b, with temperatures between 470 °C and 610 °C, the mass loss varied slightly between 5.16 wt% and 5.37 wt%. In phase II.b, the mass loss rate was slower compared to phase II.a, whose degradation corresponded to the decomposition of lignin, which occurs over a wide temperature range (160 °C to 900 °C) [54]. Lignin makes the main contributions to char formation, and due to the high aromatic content, it decomposes slowly [24]. A peak near 490 °C is observed in this stage, corresponding to the thermal degradation of cutin [6]. In the third phase (610 °C to 900 °C), the mass loss ranged from 6.22 wt% to 10.48 wt%, corresponding to the degradation of lignin and other compounds with stronger chemical bonds [55].

For PG5 under the inert atmosphere (Figure 1b), as mentioned above, the behavior is similar to PG0. Phase I occurred between ambient temperature and ~150 °C, with a mass loss between 7.31 wt% and 7.87 wt%, mainly attributed to biomass dehydration and glycerol degradation. PG5 exhibited 8.66 wt% of glycerol in the chemical composition [6]. The temperature of the main reaction area (phases II.a and II.b) for PG5 ranged between 150 °C and 590 °C. The TG curves and the maximum DTG peak moved to a higher temperature region with increasing heating rate (346 °C, 353 °C, and 358 °C for 20, 30, and 40 °C/min, respectively) without changing the thermal decomposition patterns [56]. This behavior is ascribed to limitations in heat and mass transfer within and between particles at higher heating rates [45,57]. Consequently, when the particle surface begins to degrade, the gas carries the reaction products because the internal temperature of the biomass particles is lower than the surface temperature, hindering the reaction inside the particles due to delayed heat transfer [58]. For PG5, phase II.a was developed between 150 °C and 480 °C with an average mass loss of 45.1 wt% due to the thermal decomposition of hemicellulose and cellulose (Figure 1b). Phase II.b for PG5 occurred between 480 °C and 590 °C with an average mass loss of 3.97 wt% attributed to the lignin [13] and cutin [6] degradation. The percentage of remaining material ranged from 35.86 wt% to 37.29 wt%, corresponding to biochar and ash.

**Figure 2:**
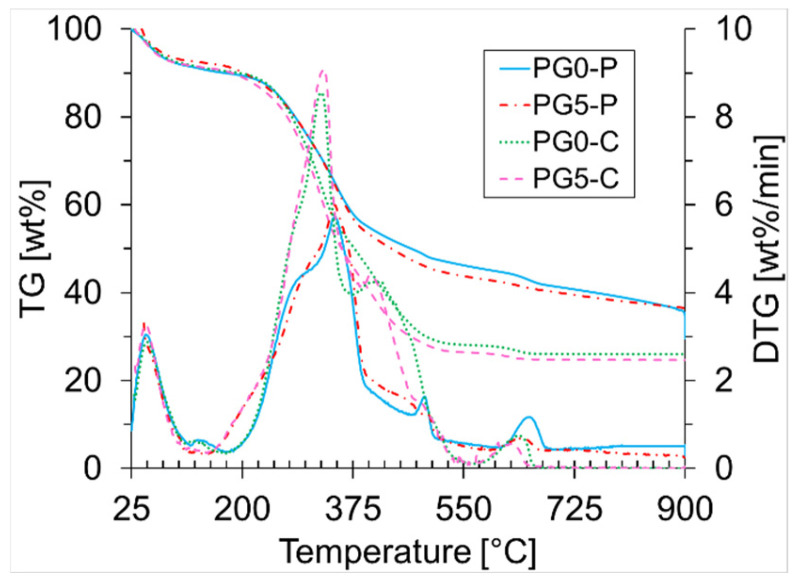
Comparison of TG and DTG curves for PG0 and PG5 under pyrolysis (P) and combustion (C) at 20 °C/min.

The TG and DTG curves for PG0 and PG5 under oxidative atmosphere are presented in Figure 1c,d, respectively. Three phases are also observed in the mass loss profile during combustion. For PG0 (Figure 1c), phase I occurred between ambient temperature and ~185 °C, corresponding to moisture evaporation (~9.85 wt%). Zone II.a (the highest oxidation rate zone) occurred between 185 °C and 370 °C, with mass loss values between 36.94 wt% and 38.79 wt% for the different heating rates. In this zone, the maximum mass loss rate occurred at the temperature of 325 °C for 20 °C/min, 331 °C for 30 °C/min, and 328 °C for 40 °C/min, and compared to the pyrolysis of PG0 (Figure 1a), this stage ended 100 °C earlier for PG0 under combustion. The lower temperatures for hemicellulose and cellulose degradation of PG0 under an oxidative atmosphere explain the higher reactivity of this biomass under combustion compared to pyrolysis. Oxidation zone II.b occurred between 370 °C and 580 °C as a result of lignin degradation (Figure 1c) and it was of higher intensity compared to pyrolysis, with a mass loss between 23.48 wt% and 25.02 wt% at different heating rates. Finally, zone III for PG0 under the oxidative atmosphere corresponded to char oxidation with a slow mass loss rate, whose mass loss was only 2.03 wt% of the initial mass. Under the oxidative atmosphere, the lignin devolatilization process is also associated with the start of char oxidation due to the mass loss rates being significantly higher (Figure 2) [22]. The material resulting from the combustion of PG0 corresponded to ash [12] with an average of 26.02 wt% of the initial mass, confirming the high ash content shown in the proximate analysis of biofuels (Table 1).

Considering PG5 under the oxidative atmosphere, Figure 1d shows the TG and DTG graphs. Like pyrolysis, the first phase describes biomass drying and glycerol decomposition, which occurs between ambient temperature and ~150 °C. Phase II.a started at a temperature of 150 °C and ended at 385 °C, due to a higher reactivity for combustion of PG5 compared to pyrolysis (Figure 1b). The higher reactivity under combustion is evident because phase II.a concluded 95 °C earlier for combustion, whose average mass loss was 47.48 wt%. The temperature of Phase II.b for PG5, under the oxidative atmosphere, was between 385 °C and 570 °C, where lignin was degraded while the char is produced. The mass loss in this phase (II.b) was 18.86 wt%; therefore, the ash content of PG5 varied between 21.98 wt% and 24.65 wt% (phase III).

Figure 2 presents the TG curves for PG0 and PG5 under pyrolysis and combustion at 20 °C/min. These graphs show a similar trend in the drying stage, as evidenced by a mass loss with a similar magnitude (~9.20 wt%). Nevertheless, for the remaining degradation process, the mass loss is faster for combustion than for pyrolysis. This is because oxidative devolatilization exhibits a more pronounced phase between temperatures of 250 °C and 370 °C, with peak degradation rates of 8.55 wt%/min at 325 °C for PG0, and 9.09 wt%/min at 329 °C for PG5. The reactions occur in a narrower temperature range and the biomass experiences a higher mass loss (~47 wt%) in comparison with the pyrolysis (~43 wt%). In the case of pyrolysis, the temperature range of the most reactive zone is between 250 °C and 470 °C, with the maximum peaks of 5.73 wt%/min (347 °C) and 6.00 wt%/min (346 °C) for PG0 and PG5, respectively. The higher reactivity of the biomass samples (PG0 and PG5) under the oxidative atmosphere can be attributed to the simultaneous degradation of less reactive lignin and the degradation of the carbonaceous structure during the combustion process. This behavior is ascribed to the easy diffusion of oxygen through the biomass due to its high porosity produced after the cellulose devolatilization. In consequence, the mass loss rate increases [59]. Similar behavior was observed by Sait et al. [26], who studied date palm seeds, stems, and leaves.

Figure 2 illustrates that the char production from the biomass samples (PG0 and PG5) is similar under the same atmospheres. Nevertheless, the behavior differs when the atmosphere varies, with an inert (N_2_) or oxidative (air) atmosphere. It is observed that under the oxidative atmosphere, less biochar is produced compared to the inert one. This behavior can be ascribed to oxygen in the air as an oxidizing agent that accelerates the devolatilization and oxidation reactions [60].

### 3.3. Reactivity and Reaction Rate

The second approach to analyze the thermal decomposition of biomass samples is based on the reactivity. The characteristic reactivity parameters of PG0 and PG5, under pyrolysis and combustion, were determined following the methodology proposed by Liu et al. [13], considering our data from Figure 2. Such parameters are presented in Table 2 for a heating rate of 20 °C/min. T_i_ and T_f_ correspond to the reaction start temperature (pyrolysis/combustion) and the end of reaction temperature, respectively. T_max_ is the temperature corresponding to the maximum reaction rate (ϒ_max_). T_f_ is defined as the temperature when the conversion rate reaches 98%, and T_i_ is also known as the initiation temperature of biomass devolatilization; this temperature is described in detail in the supplementary material of Liu et al. [13]. Differences in hemicellulose, cellulose, and lignin contents are responsible for the variation in characteristic temperatures in different biomass types [13]. The T_i_ of PG0-P is ~1.3% lower than that of PG5-P (Table 2), due to the higher amount of cellulose present in PG0, with a value of 32.2 wt% compared to 24.05 wt% for PG5 (Table 1). As cellulose pyrolyzes at low temperatures, volatile matter is more easily separated during biomass thermal degradation [13]. It is noteworthy that the biomass samples, studied herein, could diminish the reaction start temperature for pyrolysis and combustion regimes of other feedstocks that exhibit higher start temperatures if the pelletized garden waste is mixed with them.

The type of atmosphere (inert or oxidative) also affects the values of the characteristic temperatures. Table 2 shows that both biomass samples (PG0 and PG5) present a lower start reaction temperature (T_i_) under pyrolysis (225 °C and 228 °C for PG0 and PG5, respectively) compared to the start temperature under combustion for PG0 (242 °C) and PG5 (238 °C). T_max_ was higher for pyrolysis than combustion; this behavior is related to biomass reactivity, since as T_max_ increases, the biomass reactivity diminishes [61]. Here, the lowest reactivity was found for PG0 with a T_max_ of 347 °C (Table 2), followed by PG5 with a T_max_ of 346 °C, both under pyrolysis. This similar behavior, found for PG0 and PG5 under reactivity analysis, is ascribed to the similar feedstock composition of the pellet samples. As previously analyzed, PG0 and PG5 exhibited higher reactivity under combustion than under pyrolysis, with T_max_ of 325 °C for PG0, and 329 °C for PG5. Comparing the two biomass samples under combustion, PG0 exhibited higher reactivity than PG5 since the maximum reactivity was reached at a slightly lower temperature for PG0 (325 °C) compared to PG5 (329 °C).

Concerning T_f_, it is confirmed that the combustion process is faster than the pyrolysis. The combustion process was completed at a temperature of 614 °C for PG0, and 586 °C for PG5, while pyrolysis ended at temperatures of 898 °C and 858 °C for PG0 and PG5, respectively (Table 2). This behavior is ascribed to the higher reactivity found for the leaf litter pellets under an oxidative atmosphere, as described in Section 3.2. The comparison between T_f_ of PG0 and PG5, under the same atmosphere, reveals that the pyrolysis and combustion of PG5 end faster than PG0. This is attributed to the higher volatile matter content (4.4%) and lower ash content (14.7%) of PG5, enabling this material to achieve higher degradation rates [62], and consequently making the pyrolysis/combustion process finish at a low temperature (in less time).

The base temperature (T_base_) and reaction rate (R_a_) are also parameters that allow characterizing the thermal behavior of the biomass from the reactivity perspective under different atmospheres. As indicated in Table 2, the base temperature for both biomass samples was slightly higher under pyrolysis than under combustion. The base temperature for PG0-P was 219 °C, while that for PG0-C was 217 °C. The base temperature for PG5-P was 186 °C, while that for PG5-C was 183 °C. A comparison of the base temperature between the two biomass types reveals that the average temperature under pyrolysis (218 °C) is ~18% higher than the average temperature under combustion (185 °C). This confirms that PG0 and PG5 exhibit higher reactivity under combustion, as a lower base temperature is associated with lower thermal stability or higher reactivity [37].

R_a_ is related to the maximum peak of the DTG curve in units of mg/min or wt%/min, which are reached at T_max_ shown in Table 2. The reaction rate was calculated using Equation (1) for the biomass samples under inert and oxidative atmospheres. PG5 exhibits greater reactivity in pyrolysis and combustion than PG0. This is evidenced by the fact that PG5 achieved higher reactivity in inert (5%) and oxidative (6%) atmospheres than that of PG0. The higher thermal stability observed for PG0 can be attributed to its higher fixed carbon and lignin content compared to PG5 (Table 1) [51]. A comparison of the reactivity (R_a_) of the same biomass under inert and oxidative atmospheres revealed that the R_a_ value for PG0-C was ~51% higher than PG0-P. The values for combustion and pyrolysis were 0.086 min^−1^ and 0.057 min^−1^, respectively. In contrast, the reactivity of PG5-C (0.091 min^−1^) was ~52% higher than that of PG5-P (0.060 min^−1^). This result is related to a lower temperature for the maximum DTG peak under combustion (Table 2). When the maximum peaks occur at lower temperatures, this means a higher tendency to release volatile matter from the biomass structure [36]. These findings corroborate the higher reactivity of both biofuels (PG0 and PG5) during combustion, based on different analyses of the thermochemical processes for the pelletized waste garden.

**Figure A1:**
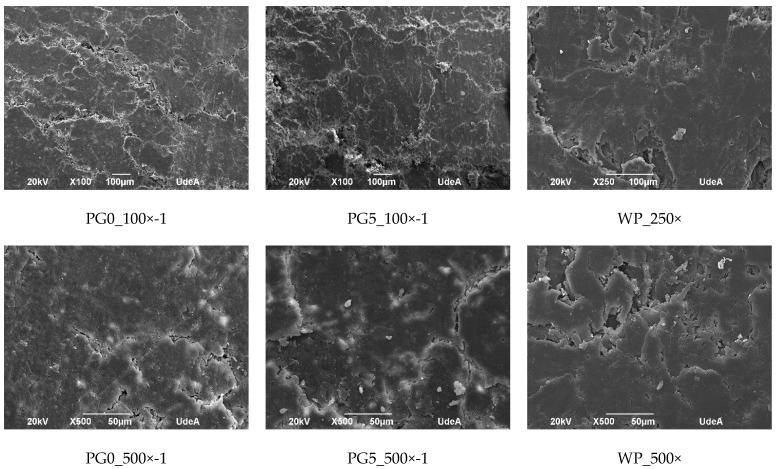
SEM images of PG0, PG5, and WP for comparison.

Figure A1 presents the surface morphology for PG0, PG5, and WP, obtained by SEM images. WP is used as a reference biofuel for comparison. The three types of biomass exhibit a compact surface structure, due to the mechanical densification treatment used to obtain pellets [6]. For PG0, a smooth surface is observed, constituted by sheets with few interstices and better mechanical interlocking; the natural agglomeration of the lignin forms the continuous layers. For PG5, a surface with some roughness is observed due to the pores and cracks caused by the bonding of lamellae, which is favored by the agglomeration of glycerol and the formation of solid lignin bridges [6]. Comparing the pictures of WP, PG0, and PG5, a similar structure is observed, which is ascribed to lignin deformation during the densification process. Nevertheless, the WP’s surface structure is smoother regarding PG0 and PG5. This is attributed to the higher lignin content of WP (Table 1) that promotes densification [37]. Given the similarity in surface morphology and physicochemical properties between these biomass samples (as shown in Table 1), in future studies, it is important to investigate the thermal behavior of WP and PG0-PG5 blends. This will help to assess their reactivity and potential use as solid biofuels enhancing the biomass energy recovery capacity because wood [63] as well as fallen leaves could be used as feedstock for bioenergy projects.

### 3.4. Pyrolysis and Combustion Kinetics

**Figure 3:**
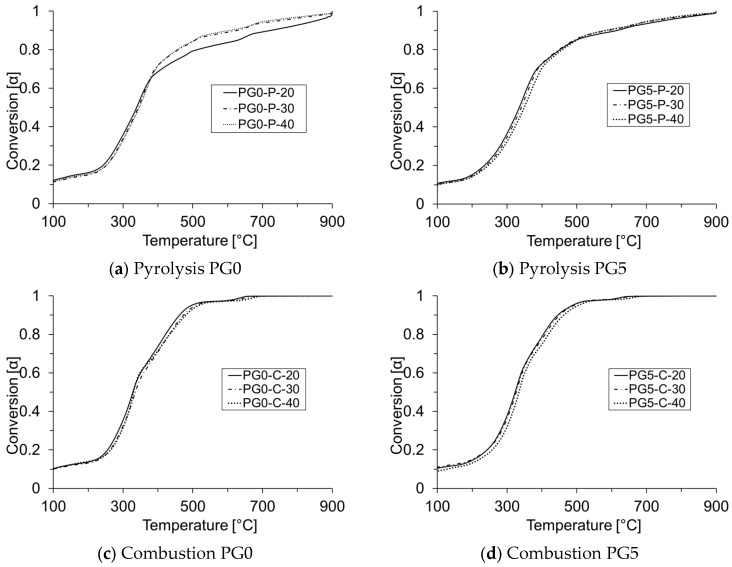
Degree of conversion (α) at the different heating rates (20, 30, and 40 °C/min) for PG0 and PG5 under pyrolysis (P) and combustion (C).

Figure 3a–d shows the variation of conversion (α) for both biomass as a function of temperature, heating rates (20, 30, and 40 °C/min), and atmosphere type (inert or oxidative). The calculation of α was performed using Equation (2). Here, the activation energy and the pre-exponential coefficient were calculated considering the range 0.2 ≤ α_i_ ≤ 0.8, which corresponds to the range in which the highest devolatilization/oxidation of biomass occurs and where the linear regression coefficient (R^2^) reached values higher than 0.9 ensuring repeatability and statistical reliability [11]. These considerations guarantee that the degree of conversion exhibits an appropriate degree of linearity. Figure 3a–d illustrates that the degree of conversion increases as the reaction temperature rises. Moreover, it is shown that the conversion rate for both biomass follows the same profile, in pyrolysis as in combustion. The α profile is not significantly affected by the variation in heating rate. As previously stated in Section 3.2, the biomass conversion rate is affected by temperature, thus three main reaction zones are evidenced for both pyrolysis and combustion regimes, and the zones are a function of the graph’s slope. These zones are associated with the thermal decomposition of glycerol, hemicellulose, cellulose, cutin, and lignin [64].

**Figure A2:**
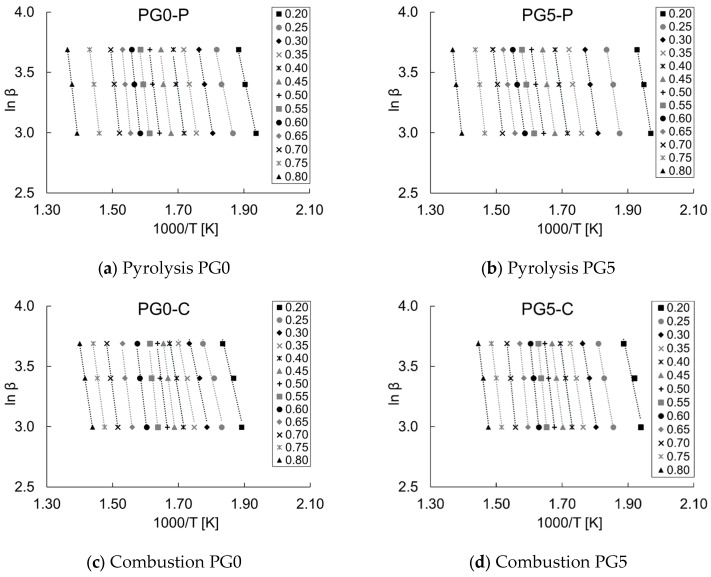
Regression lines at different values of α used to calculate $E_{\alpha }$ by the FWO method for PG0 and PG5 under pyrolysis (P) and combustion (C).

**Figure A3:**
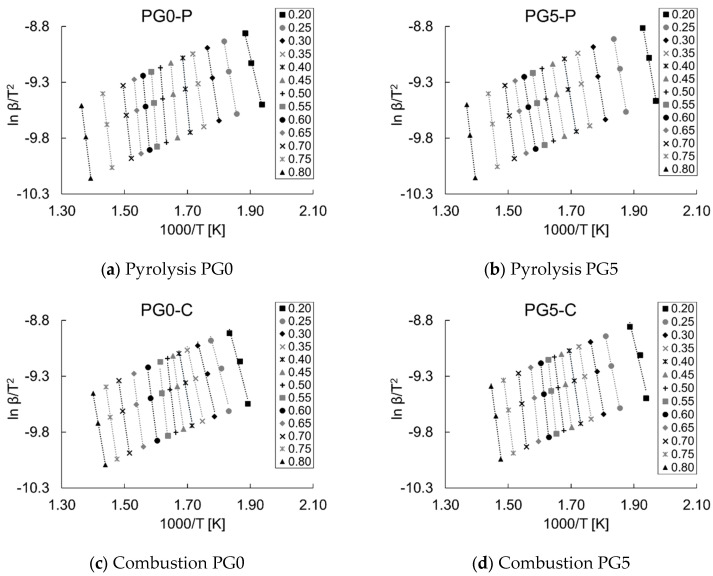
Regression lines at different values of α used to calculate $E_{\alpha }$ by the KAS method for PG0 and PG5 under pyrolysis (P) and combustion (C).

**Figure A4:**
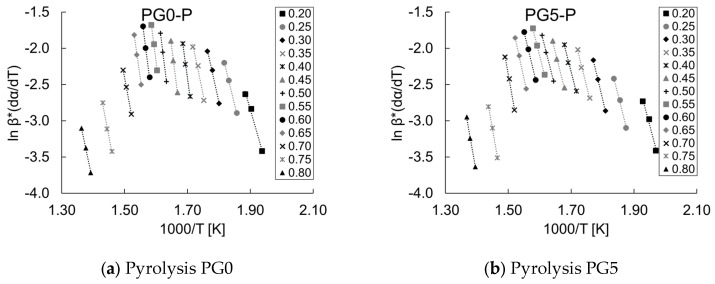

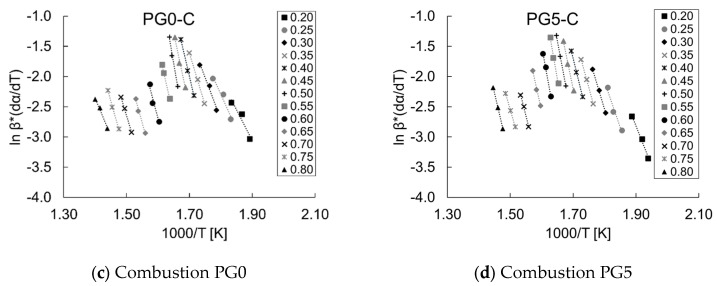
Regression lines at different values of α used to calculate $E_{\alpha }$ by the Friedman method for PG0 and PG5 under pyrolysis (P) and combustion (C).

Figure A2 depicts the isoconversional lines or regression lines within the α_i_ range, which are necessary for the calculation of the activation energy (E_α_, kJ/mol) by the Flynn–Wall–Ozawa (FWO) method, as outlined in Equation (6). Figure A3 presents the isoconversional lines for the Kissinger–Akahira–Sunose (KAS) method, which were calculated using Equation (7). Finally, Figure A4 shows the regression lines for the Friedman method, that were calculated following Equation (8). The variation of the findings of the FWO, KAS, and Friedman analyses is less than 5%. Therefore, the proximity of the outcomes suggests uniformity, reliability, and applicability of the three methods for the calculi of kinetic parameters [42].

Table 3 shows the E_α_, A_α_, and R^2^ values for PG0 and PG5 under pyrolysis and combustion as a function of α. In general, the activation energy increased up to a certain degree of conversion and then decreased, as Kumar and Mohanty [11] and Islamova et al. [65] also showed by studying the thermal decomposition of seeds. This is because weaker bonds decompose at moderate energy and lower temperature, whereas degradation of stronger bonds needs more energy and higher temperature [13]. The average values of E_α_ obtained for PG0-P from the FWO, KAS, and Friedman models were 105.23 kJ/mol, 105.33 kJ/mol, and 108.82 kJ/mol, respectively, while PG5-P reached lower values for E_α_, with 96.16 kJ/mol (FWO), 94.84 kJ/mol (KAS), and 99.46 kJ/mol (Friedman). Thereby, PG5 biomass is more reactive than PG0 under pyrolysis. The activation energy is defined as the minimum amount of energy required to initiate a reaction. Therefore, low values of E_α_ mean that the reactants reacted more easily at lower temperatures [53,66]. This result is consistent with the TG and DTG analysis (Section 3.2), which demonstrated that the devolatilization under pyrolysis of PG5 occurs at lower temperatures in contrast to PG0.

A comparison of the average E_α_ under combustion (Table 4) for PG0 and PG5 reveals that the variation of this parameter is less than 1%, with values of 90.70 kJ/mol and 90.29 kJ/mol, respectively. This finding agrees with the observations from both biofuels’ surface morphology and physical properties (Section 3.3). Against other biomass types such as sunflower seeds with an activation energy under combustion of 175.39 kJ/mol [65], while the flax straw biomass reached an E_α_ of 215.5 kJ/mol [67]. The PG0-C and PG5-C simples exhibit lower thermal stability and require less energy to activate the oxidation chemical reaction [65].

When comparing the activation energy for the two-atmosphere types studied here (inert/oxidative), it was found that E_α_ is ~18% higher for PG0-P concerning PG0-C. Similarly, E_α_ of PG5-P is ~7% higher than that of PG5-C. Therefore, the higher reactivity of PG0 and PG5 under combustion is corroborated; likewise, the result is consistent with the analysis of T_max_ and T_f_ in Section 3.3.

For PG0 and PG5 under the oxidative atmosphere, the highest E_α_ was reached for α values between 0.55 and 0.60, which coincides with the temperatures at which the maximum thermal degradation rate is reached in the DTG diagram (Figure 1c,d). Therefore, the combined decomposition of cellulose and lignin in the devolatilization stage requires a high E_α_ [24]. E_α_ for PG0 and PG5 under pyrolysis and combustion, characterized herein (Table 4), agree with the values reported in the literature for agricultural residues (75–130 kJ/mol) [68,69], and grassy biomass (*Penissetum glaucum*), which reached values under pyrolysis between 37.18–132.39 kJ/mol by the FWO method, and 25.56–129.06 kJ/mol by the KAS method. For combustion regimes, the grassy biomass (*Penissetum glaucum*) reached E_α_ values between 32.53–77.98 kJ/mol when analyzed using the FWO method, and between 20.45–72.17 kJ/mol when analyzed using the KAS method [14]. E_α_ of PG0 and PG5 was lower when compared to other densified biomass, such as the case of patula wood pellets (WP), whose average value of E_α_ under pyrolysis was 124.38 kJ/mol [25]. Cai et al. [45] reported an average of E_α_ under N_2_ of 206.66 kJ/mol for tea leaves. E_α_ of date palm seeds stems and leaves ranged between 9.7–43.6 kJ/mol under pyrolysis, and values between 9.04–30.95 kJ/mol under combustion [26].

**Figure A5:**
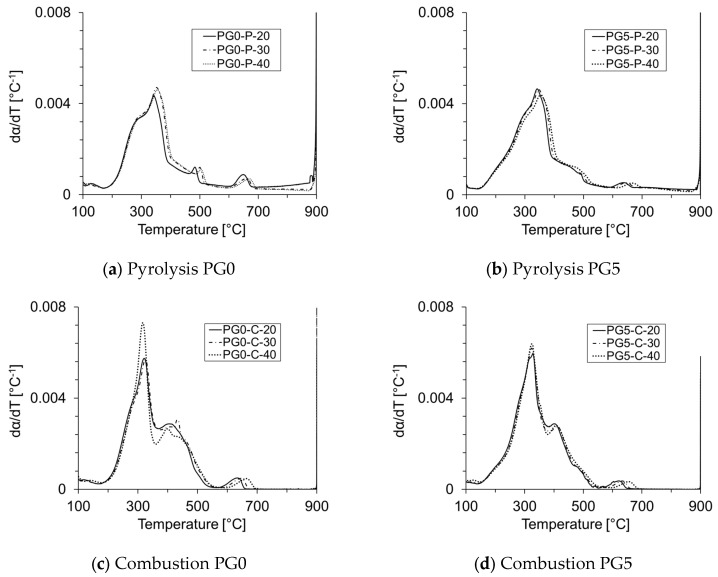
Curves dα/dT at the different heating rates used to calculate Aα for PG0 and PG5 under pyrolysis (P) and combustion (C).

The pre-exponential factor (A_α_, s^−1^) for PG0 and PG5 under pyrolysis and combustion were calculated using Equation (9). Figure A5 depicts the dα/dT vs. T curves employed to determine the peak temperature (T_m_, K). A_α_ exhibited a numerical variation as a function of α, as shown in Table 3. This variation followed a similar trend observed in E_α_, due to the correlation between these two variables as outlined in Equation (9). For PG0 under pyrolysis, A_α_ reached values from 2.13 × 10^2^ s^−1^ to 1.87 × 10^10^ s^−1^ by the FWO method. PG5, under the inert atmosphere, ranged between 4.28 × 10^3^ s^−1^ and 2.04 × 10^11^ s^−1^ by the FWO method. The fluctuations in A_α_ account for the varied lignocellulosic composition of the biofuel and the complex reactions occurring during the thermal decomposition of the material [70]. Values of A_α_ less than 10^4^ s^−1^ are related to low E_α_, in this case, <80 kJ/mol, which represents hemicellulose degradation [48]. Values of A_α_ < 10^9^ s^−1^ mainly indicate a surface reaction, whereas if the reactions do not depend on the surface area, A_α_ reflects a closed activated complex (tight binding). Otherwise, A_α_ ≥ 10^9^ s^–1^ indicates a simple activated complex [48], in which there is a higher molecular collision rate for monomolecular decomposition within the solid phase, as is the case for the decomposition of cellulose and hemicellulose [24]. In combustion, A_α_ values of PG0 ranged between 1.59 × 10^2^ s^−1^ and 2.12 × 10⁸ s^−1^ by the FWO method, while PG5 reached values between 2.91 × 10^2^ s^−1^ and 1.37 × 10⁸ s^−1^ by the FWO method (Table 3).

The average values for A_α_ for the three model-free isoconversional methods are presented in Table 4. Under pyrolysis, the average values of A_α_ were 3.67 × 10^9^ s^−1^ and 3.18 × 10^9^ s^−1^ for PG0 and PG5, respectively. For the combustion regime, the average values of A_α_ for PG0 and PG5 were 3.7 × 10^8^ s^−1^ and 4.36 × 10^7^ s^−1^, respectively. The results (A_α_) found here are consistent with those reported in the literature. Maia and De Morais [48] reported an A_α_ between 3.80 × 10^0^ s^−1^ and 2.80 × 10^12^ s^−1^ for red bell pepper residues under an oxidative atmosphere, with E_α_ ranging from 29.49 to 123.88 kJ/mol. For tea leaves under combustion, Cai et al. [45] found an A_α_ between 1.27 × 10^9^ s^−1^ and 2.77 × 10^24^ s^−1^ (FWO method), for E_α_ of 102.65 kJ/mol and 268.44 kJ/mol, respectively. On the other hand, Gutierrez et al. [25] reported A_α_ values of 5.05 × 10^7^ s^−1^ (E_α_, 118.79 kJ/mol) and 3.50 × 10^8^ s^−1^ (E_α_, 128.67 kJ/mol) for WP under pyrolysis kinetics using FWO method. Kumar and Mohanty [11] characterized seeds of *Manilkara zapota* (CK), *Delonix regia* (SG), and *Cascabela thevetia* (SK) under pyrolysis by the KAS method and reported average values for A_α_ of 1.56 × 10^9^ s^−1^ for CK, 4.29 × 10^9^ s^−1^ for SG, and 4.18 × 10^9^ s^−1^ for SK, with average E_α_ of 131.73 kJ/mol, 142.52 kJ/mol and 151.74 kJ/mol, respectively.

Considering the heating value, the high volatile matter content, and the low activation energy of the fallen leaf pellets, it is worth mentioning that these pellets are a source derived from green waste with the potential to produce energy by thermochemical conversion processes. The thermal behavior assessment and kinetic data at different heating rates of leaf litter pellets under inert and oxidative atmospheres could be considered initial technical feasibility. These results are the starting point for future works such as modeling thermochemical processes, as well as the design and development of thermochemical conversion applications. Therefore, this work promotes the use of leaf litter as solid biofuel and provides a new roadmap for waste management policies contributing to the reduction in greenhouse gas emissions.

## 4. Conclusions

The fallen leaf pellets were characterized by thermogravimetric analyses to assess their reactivity as solid biofuels under thermochemical processes (pyrolysis and combustion), whereby the following conclusions can be drawn.

(1) The practical implications of using glycerol-bound garden waste pellets in industrial thermochemical processes are worth mentioning. The pelletized green wastes (fallen tree leaves) can be used as solid biofuels. PG5 is more reactive compared to PG0 because the glycerol added as a binder to the pellets increased the volatile matter content, while the fixed carbon content decreased. Consequently, the thermal degradation of PG5 is favored regarding PG0.

(2) The thermogravimetric (TG) and derivative thermogravimetric (DTG) profiles for pyrolysis and combustion of PG0 and PG5 exhibited three phases throughout the thermal decomposition, namely drying or dehydration, devolatilization, and carbonization. For PG5, the drying zone was accompanied by glycerol degradation. In the devolatilization zone, besides the degradation of hemicellulose, cellulose, and a fraction of lignin, the cutin was also thermally degraded.

(3) The lowest temperatures for the maximum peak in the DTG curve were observed in an oxidative atmosphere, indicating that PG0 and PG5 exhibited higher reactivity under combustion (oxidative atmosphere) than under pyrolysis (inert atmosphere). This was ascribed to the oxygen in the air, which can easily diffuse into the biomass after cellulose devolatilization. Consequently, T_f_ indicated a higher reaction rate for the studied biofuels under combustion, as this thermochemical process was completed at 614 °C and 586 °C for PG0 and PG5, respectively. Whereas, during pyrolysis, T_f_ was 898 °C for PG0, and 858 °C for PG5.

(4) E_α_ for both feedstocks is relatively low, with average values of 106.46 kJ/mol (PG0-P), 96.82 kJ/mol (PG5-P), 90.70 kJ/mol (PG0-C), and 90.29 kJ/mol (PG5-C). Therefore, these biomass samples require low energy input to start the pyrolysis and combustion reactions, which favors the thermochemical transformation process. The properties of the pellets studied herein are comparable with other biomasses, such as pine wood pellets, which have a wide range of applications to produce energy and biochar through thermochemical processes. Therefore, the potential for using fallen leaves as an energy source is worth highlighting.

(5) Finally, the coherence between the three methods (TG-DTG analyses, TG-based reactivity, and reaction kinetics) used to evaluate the reactivity of biomasses is worth noting, since the behavior and the trends are similar. Nevertheless, the choice of one of the methods depends on the research needs, such as evaluating the reactivity or thermal stability of biomasses, determining decomposition rates, or the kinetics of reactions for reactor design, and modeling of thermochemical processes. Herein, the technical feasibility of pelletizing fallen leaf trees with glycerol as a binder and their kinetic behavior under pyrolysis and combustion are assessed. Nevertheless, in future works, the pelletization process at an industrial scale must be addressed, through experimental and/or modeling processes, for economic and energy feasibility assessment.

## Figures and Tables

**Table 2:** Characteristic parameters, base temperature, and reactivity for PG0 and PG5 under pyrolysis and combustion.

Biomass	T_i_ (°C)	T_max_ (°C)	T_f_ (°C)	ϒ_max_ (wt%/min)	T_base_ [°C]	R_a_ [min^−1^]
PG0-P	225	347	898	5.73	219	0.057
PG5-P	228	346	858	6.00	186	0.060
PG0-C	242	325	614	8.55	217	0.086
PG5-C	238	329	586	9.09	183	0.091

**Table 3:** Results of E_α_ [kJ/mol], A_α_ [s^−1^], and R^2^, for PG0 and PG5 under pyrolysis and combustion.

Atmosphere	α	PG0/Model									PG5/Model								
		FWO			KAS			Friedman			FWO			KAS			Friedman		
		E_α_	A_α_	R^2^	E_α_	A_α_	R^2^	E_α_	A_α_	R^2^	E_α_	A_α_	R^2^	E_α_	A_α_	R^2^	E_α_	A_α_	R^2^
N_2_	0.20	53.17	2.13 × 10^2^	0.997	51.38	1.46 × 10^2^	0.997	65.12	2.57 × 10^3^	0.985	67.86	4.28 × 10^3^	0.991	66.99	3.58 × 10^3^	0.990	70.16	7.43 × 10^3^	0.976
	0.25	58.45	6.38 × 10^2^	0.982	57.63	5.38 × 10^2^	0.982	62.58	1.50 × 10^3^	0.997	75.41	2.03 × 10^4^	0.988	74.49	1.68 × 10^4^	0.987	77.81	3.58 × 10^4^	0.993
	0.30	73.35	1.39 × 10^4^	1.000	72.54	1.17 × 10^4^	1.000	80.86	6.42 × 10^4^	0.999	79.33	4.54 × 10^4^	0.999	78.34	3.71 × 10^4^	0.999	84.71	1.46 × 10^5^	1.000
	0.35	83.34	1.06 × 10^5^	0.999	82.59	9.13 × 10^4^	0.999	93.38	8.13 × 10^5^	0.989	80.28	5.53 × 10^4^	0.981	78.96	4.21 × 10^4^	0.978	82.55	9.42 × 10^4^	0.992
	0.40	92.47	6.70 × 10^5^	0.957	93.41	8.10 × 10^5^	0.956	103.34	5.99 × 10^6^	0.968	84.56	1.33 × 10^5^	0.987	83.17	9.99 × 10^4^	0.985	82.88	1.01 × 10^5^	0.993
	0.45	100.97	3.72 × 10^6^	0.962	102.28	4.84 × 10^6^	0.961	110.34	2.44 × 10^7^	0.975	87.57	2.45 × 10^5^	0.993	86.06	1.80 × 10^5^	0.992	86.37	2.05 × 10^5^	0.997
	0.50	110.07	2.31 × 10^7^	0.970	111.83	3.28 × 10^7^	0.970	112.50	3.75 × 10^7^	0.978	89.41	3.56 × 10^5^	0.993	87.81	2.57 × 10^5^	0.992	86.58	2.15 × 10^5^	0.998
	0.55	118.92	1.35 × 10^8^	0.983	121.03	2.07 × 10^8^	0.983	112.59	3.82 × 10^7^	0.976	90.79	4.73 × 10^5^	0.997	89.00	3.28 × 10^5^	0.996	89.00	3.51 × 10^5^	1.000
	0.60	131.80	1.77 × 10^9^	0.984	132.77	2.15 × 10^9^	0.984	140.02	9.06 × 10^9^	0.981	94.22	9.49 × 10^5^	0.997	92.33	6.46 × 10^5^	0.996	95.73	1.37 × 10^6^	1.000
	0.65	143.65	1.87 × 10^10^	0.991	144.76	2.32 × 10^10^	0.990	150.18	6.79 × 10^10^	0.994	100.47	3.36 × 10^6^	0.998	98.67	2.33 × 10^6^	0.998	108.41	1.76 × 10^7^	0.999
	0.70	137.17	5.17 × 10^9^	0.999	135.75	3.90 × 10^9^	1.000	127.21	7.13 × 10^8^	1.000	123.60	3.51 × 10^8^	0.996	122.73	2.95 × 10^8^	0.995	137.16	5.49 × 10^9^	0.995
	0.75	129.59	1.15 × 10^9^	0.997	130.00	1.25 × 10^9^	0.997	130.82	1.47 × 10^9^	0.993	132.67	2.16 × 10^9^	0.998	131.66	1.76 × 10^9^	0.998	142.27	1.52 × 10^10^	0.998
	0.80	135.06	3.43 × 10^9^	0.995	133.25	2.39 × 10^9^	0.997	125.75	5.38 × 10^8^	0.998	143.94	2.04 × 10^10^	0.999	142.69	1.59 × 10^10^	0.999	149.39	6.23 × 10^10^	0.997
	**Average**	**105.23**	**2.34 × 10^9^**	**0.986**	**105.33**	**2.55 × 10^9^**	**0.986**	**108.82**	**6.13 × 10^9^**	**0.987**	**96.16**	**1.76 × 10^9^**	**0.994**	**94.84**	**1.38 × 10^9^**	**0.993**	**99.46**	**6.39 × 10^9^**	**0.995**
Air	0.20	49.86	1.59 × 10^2^	0.965	47.68	9.81 × 10^1^	0.958	45.10	6.08 × 10^1^	0.915	53.41	2.91 × 10^2^	0.941	51.62	1.98 × 10^2^	0.931	57.45	7.52 × 10^2^	0.991
	0.25	52.17	2.64 × 10^2^	0.958	49.77	1.56 × 10^2^	0.949	53.27	3.68 × 10^2^	0.946	59.05	9.79 × 10^2^	0.995	57.69	7.32 × 10^2^	0.995	61.59	1.81 × 10^3^	0.943
	0.30	57.40	8.26 × 10^2^	0.963	55.00	4.90 × 10^2^	0.956	65.35	5.03 × 10^3^	0.981	66.48	4.77 × 10^3^	0.998	65.28	3.69 × 10^3^	0.997	72.31	1.75 × 10^4^	0.999
	0.35	65.35	4.61 × 10^3^	0.971	63.15	2.87 × 10^3^	0.966	84.03	2.69 × 10^5^	0.998	71.39	1.35 × 10^4^	1.000	70.28	1.07 × 10^4^	1.000	79.00	7.12 × 10^4^	0.999
	0.40	77.82	6.62 × 10^4^	0.988	76.07	4.57 × 10^4^	0.986	109.89	6.08 × 10^7^	0.997	77.78	5.17 × 10^4^	0.999	76.88	4.28 × 10^4^	0.999	88.96	5.68 × 10^5^	0.993
	0.45	98.25	4.95 × 10^6^	1.000	97.38	4.12 × 10^6^	1.000	122.08	7.59 × 10^8^	0.988	85.92	2.84 × 10^5^	0.995	85.37	2.54 × 10^5^	0.995	105.92	1.89 × 10^7^	0.985
	0.50	106.47	2.76 × 10^7^	0.969	106.56	2.81 × 10^7^	0.966	133.70	8.36 × 10^9^	0.982	93.54	1.39 × 10^6^	0.989	93.33	1.33 × 10^6^	0.988	118.74	2.63 × 10^8^	0.989
	0.55	127.29	2.12 × 10^9^	0.950	127.61	2.26 × 10^9^	0.945	115.27	1.82 × 10^8^	0.999	106.21	1.92 × 10^7^	0.975	106.66	2.10 × 10^7^	0.974	121.57	4.67 × 10^8^	0.964
	0.60	113.70	1.26 × 10^8^	0.970	113.02	1.09 × 10^8^	0.967	103.85	1.69 × 10^7^	0.927	108.62	3.15 × 10^7^	0.982	109.04	3.43 × 10^7^	0.981	118.84	2.67 × 10^8^	0.999
	0.65	113.04	1.10 × 10^8^	0.962	111.97	8.77 × 10^7^	0.958	99.14	6.34 × 10^6^	0.985	115.77	1.37 × 10^8^	1.000	116.33	1.54 × 10^8^	1.000	100.65	6.33 × 10^6^	0.972
	0.70	107.79	3.68 × 10^7^	0.994	105.96	2.51 × 10^7^	0.993	96.58	3.73 × 10^6^	0.999	114.12	9.81 × 10^7^	0.997	114.30	1.02 × 10^8^	0.997	91.49	9.52 × 10^5^	1.000
	0.75	107.63	3.57 × 10^7^	0.998	105.35	2.21 × 10^7^	0.997	104.05	1.79 × 10^7^	0.995	101.75	7.67 × 10^6^	1.000	100.72	6.20 × 10^6^	0.999	84.92	2.45 × 10^5^	0.992
	0.80	98.92	5.79 × 10^6^	1.000	95.80	3.00 × 10^6^	1.000	73.82	3.07 × 10^4^	0.983	105.60	1.70 × 10^7^	1.000	104.45	1.34 × 10^7^	0.999	108.16	3.01 × 10^7^	0.996
	**Average**	**90.44**	**1.90 × 10^8^**	**0.976**	**88.87**	**1.95 × 10^8^**	**0.972**	**92.78**	**7.24 × 10^8^**	**0.976**	**89.20**	**2.41 × 10^7^**	**0.99**	**88.61**	**2.56 × 10^7^**	**0.99**	**93.05**	**8.11 × 10^7^**	**0.99**

**Table 4:** Average values of E_α_ and A_α_ for the three model-free isoconversional methods for PG0 and PG5 under pyrolysis and combustion.

Biomass	E_α_ [kJ/mol]		A_α_ [s^−1^]	
	Pyrolysis	Combustion	Pyrolysis	Combustion
PG0	106.46	90.70	3.67 × 10^9^	3.70 × 10^8^
PG5	96.82	90.29	3.18 × 10^9^	4.36 × 10^7^

## Data Availability

The original contributions presented in this study are included in the article. Further inquiries can be directed to the corresponding author.

## Abbreviations

α	Mass conversion fraction
βj	Heating rate
ϒmax	Maximum reaction rate
Aα	Pre-exponential factor
C	Combustion
DTG	Differential thermogravimetry
Eα	Activation energy
FC	Fixed carbon
FWO	Flynn–Wall–Ozawa
ICTAC	International Confederation for Thermal Analysis and Calorimetry
KAS	Kissinger–Akahira–Sunose
LHV	Lower heating value
m0	Initial mass of the sample
m∞	Final mass of the sample
MSW	Municipal solid waste
mt	Mass of the sample at a time t
P	Pyrolysis
PG0	Pellets without glycerol
PG5	Pellets with 5 wt% glycerol
R2	Linear regression coefficient
R	Universal gas constant
Ra	Reaction rate
SEM	Scanning Electron Microscopy
t	Time
Tαi	Conversion temperature at a specified αi
Tb	Base temperature
Tf	End of reaction temperature
TG	Thermogravimetry
Ti	Reaction start temperature
Tm	Temperature corresponding to the maximum point of the plot dα/dT vs. T
Tmax	Temperature corresponding to the maximum reaction rate
UdeA	University of Antioquia
VM	Volatile matter
WP	Wood pellets
